# Safe patient transport for COVID-19

**DOI:** 10.1186/s13054-020-2828-4

**Published:** 2020-03-18

**Authors:** Mei Fong Liew, Wen Ting Siow, Ying Wei Yau, Kay Choong See

**Affiliations:** 1 Division of Respiratory and Critical Care Medicine, University Medicine Cluster, National University Health System, 1E Kent Ridge Rd, Singapore, 119228 Singapore; 2 Fast and Chronic Programmes, Alexandra Hospital, National University Health System, Singapore, Singapore; 3 Emergency Medicine Department, National University Hospital, National University Health System, Singapore, Singapore

Dear Editor,

Although COVID-19 has not been officially labelled as a pandemic yet, the global burden of disease is significant and continues to rise. The virus has a high human-to-human transmissibility via airborne, droplet and contact routes [1]. Patient numbers can surge, and hospitals should be ready not just with the infrastructure, but also staff to be familiar with workflows. Kain and Fowler [2] have eloquently detailed influenza pandemic preparations for hospitals and intensive care units, and we feel the principles described in the article are relevant to COVID-19. Staff must consider patient transfers in between wards, as COVID-19 patients are admitted in isolation facilities to contain infected cases and to avoid nosocomial spread [1].

Infectious cases may be intentionally brought out of isolation rooms for various reasons. Intra-hospital transfer may be required from emergency departments to the wards, from the general floor to the intensive care unit and from the wards to radiology suites. Inter-hospital transfer may be required for extracorporeal membrane oxygenation (ECMO) if patients with COVID-19 develop severe acute respiratory distress syndrome within hospitals with only basic ventilation facilities. During episodes of patient transport outside of isolation, potential breaches of infection control can occur. At the same time, when COVID-19 patients turn ill during transport, their management is exceptionally challenging as accompanying staff would be wearing cumbersome personal protective equipment (PPE) [3].

Mitigating the spread of COVID-19 is a national priority in Singapore [4], and part of this effort involves planning and conducting safe patient transport for suspected or confirmed cases. HCWs who handle the transport of COVID-19 patients must consider the following principles (see Table 1): firstly, early recognition of the deteriorating patient; secondly, HCW safety; thirdly, bystander safety; fourthly, contingency plans for medical emergencies during transport; fifthly, post-transport decontamination. Specific action steps require designated zones for transport [5], sufficient supplies of PPE, staff training and support personnel like security officers and cleaning crews. Powered air-purifying respirators add a layer of safety on top of N95 respirators [3] and should be used if possible for high-risk cases, such as those requiring ambulance transport to ECMO centres.

**Table 1:** Patient transport issues and solutions for COVID-19

	Intra-hospital transport		Inter-hospital transport
	Transport from EMD to GW or ICU; transport from GW to ICU	Transport for radiology scans	For advanced ICU services, e.g. ECMO
Patient safety	• Early transfer of deteriorating cases to ICU	• To minimise need for scans, e.g. using bedside ultrasound	• Early transfer of deteriorating cases • Clear thresholds for transfer and workflows for non-ECMO centres
	• For deteriorating patients, to assess the need for intubation prior to transport • To be accompanied by at least a doctor and a nurse who are able to handle emergencies during transport • Continuous monitoring of parameters (blood pressure, pulse rate, pulse oximetry) • Continuous end-tidal CO2 monitoring in intubated patients • Transport monitor should be equipped with defibrillation function or else a separate defibrillator is needed		
Safety of HCW and transport staff	• All transport staff should be mask-fitted for N95 respirators • All transport staff to don full PPE prior to transport • To put on surgical mask for patient during transport • To avoid using open breathing circuits, or high-flow nasal oxygenation and non-invasive positive pressure during transport • To add on HEPA filters to endotracheal tubes if bagging is required via BVM • To add on HEPA filters to expiratory limbs of the breathing circuits for ventilators • Avoid unnecessary breathing circuit disconnection during transport • Scans to be performed at the end of the day if possible, to allow for terminal cleaning of radiology		• All transport staff should be mask-fitted for N95 respirators and trained to use PAPRs • All transport staff to don full PPE and PAPRs prior to transport • To bring along spare battery packs for PAPRs • To add on HEPA filters to endotracheal tubes if bagging is required via BVM • To add on HEPA filters to expiratory limbs of the breathing circuits for ventilators • Minimise endotracheal tube disconnections during transport • To wind down ambulance windows if possible
Bystander safety	• To use a pre-planned dedicated transport route to each destination • Security team to lead and ensure clearance of bystanders for the entire designated route ahead of transport team. Security team should wear surgical masks		
Rescue and contingency plans during transport	• To assess the need for intubation prior to transport. Intubation is best done in ICU under controlled settings with the intubating physician wearing PPE and using a PAPR • Prepare transport equipment and drugs in anticipation of medical emergencies, such as sudden cardiovascular collapse or hypotension • Gentle bagging by BVM to reduce aerosolization in the event of worsening hypoxemia. BVM should be fitted with HEPA filter		
Post-transport decontamination	• Dedicated housekeeping team in PPE to perform terminal cleaning of dedicated route and elevator right after transport • Staff to doff PPE appropriately after transport		• Dedicated housekeeping team in PPE to perform terminal cleaning of dedicated route and elevator right after transport • Staff to doff PAPRs and PPE at destination after transport • PAPRs to be wiped down and disinfected using alcohol wipes • Staff to don new PPE for the return journey prior to embarking on the same ambulance • Staff to doff PPE in the nearest clinical area, for example ambulance bay, upon arrival • Terminal cleaning of ambulance upon arrival when back at primary hospital

*BVM* bag-valve-mask, *CO2* carbon dioxide, *ECMO* extracorporeal membrane oxygenation, *EMD* emergency, *GW* general ward, *HEPA* high-efficiency particulate air, *ICU* intensive care unit, *PAPR* powered air-purifying respirator, *PPE* personal protective equipment

Given the continued global spread of COVID-19, we expect that more hospitals will need to deal with this disease. Haphazard transport of infected cases leading to nosocomial spread can stymie efforts to break the chains of transmission. We hope that our suggestions can aid others in ensuring safe patient transport for COVID-19 and reduce nosocomial spread.

## Data Availability

Not applicable.

## References

[1] Li Q, Guan X, Wu P, Wang X, Zhou L, Tong Y, et al. Early transmission dynamics in Wuhan, China, of novel coronavirus-infected pneumonia. N Engl J Med. 2020. 10.1056/NEJMoa2001316. [Epub; ahead of print]. 10.1056/NEJMoa2001316 PMC7121484 31995857

[2] Kain T, Fowler R (2019). Preparing intensive care for the next pandemic influenza. Crit Care.

[3] Wax RS, Christian MD. Practical recommendations for critical care and anesthesiology teams caring for novel coronavirus (2019-nCoV) patients. Can J Anaesth. 2020. 10.1007/s12630-020-01591-x. [Epub; ahead of print]. 10.1007/s12630-020-01591-x PMC7091420 32052373

[4] Wong JEL, Leo YS, Tan CC. COVID-19 in Singapore-current experience: critical global issues that require attention and action. JAMA. 2020. 10.1001/jama.2020.2467. [Epub; ahead of print]. 10.1001/jama.2020.2467 32077901

[5] Pan L, Wang L, Huang X. How to face the novel coronavirus infection during the 2019-2020 epidemic: the experience of Sichuan Provincial People’s Hospital. Intensive Care Med. 2020. 10.1007/s00134-020-05964-0. [Epub; ahead of print]. 10.1007/s00134-020-05964-0 PMC7079935 32072300

